# Applications of large‐scale artificial intelligence models in bioinformatics

**DOI:** 10.1002/qub2.70026

**Published:** 2025-12-22

**Authors:** Mingjing Li, Qichen Shang, Ziyang Dong, Zhixuan You, Le Zhang, Ming Xiao

**Affiliations:** ^1^ College of Computer Science Sichuan University Chengdu China

**Keywords:** artificial intelligence, bioinformatics, large‐scale language models, large‐scale multimodal models, large‐scale vision models

## Abstract

Large‐scale artificial intelligence (AI) models can mine potential patterns from massive amounts of data and provide more accurate analyses. This capability has enabled its gradual application in various areas of bioinformatics. However, few reviews have comprehensively summarized the applications of different types of large‐scale AI models in key areas of bioinformatics. Therefore, we first introduce the concept of large‐scale AI models and classify them into three types. Second, we summarize the key methods, applications, and resources of these three types of bioinformatics models. Finally, we discuss challenges and directions for future research. This review provides researchers with a comprehensive perspective to better understand the applications of large‐scale AI models in bioinformatics.

## 1 INTRODUCTION

High‐throughput technologies generate large‐scale and complex omics data that challenge classical bioinformatics methods in terms of data management and interpretation [1]. In this context, with the rapid development of artificial intelligence (AI) technologies, various classical machine learning (ML) and deep learning (DL) AI methods have been widely applied in bioinformatics, significantly improving the efficiency and accuracy of bioinformatics algorithms [2, 3, 4, 5]. For instance, self‐supervised learning techniques can enhance the accuracy of protein structure prediction [6]; DL enables effective prediction of single‐cell deoxyribonucleic acid (DNA) methylation states [7, 8]; and variational autoencoders [9] have been applied to biomolecular design.

Despite the significant achievements of AI methods in bioinformatics, they still face three major challenges. First, the performance improvement of these methods relies heavily on a large amount of high‐quality annotated data [10]. For instance, deep neural networks are often ineffective when the number of annotated proteins is low. Correctly integrating various data types and annotations also poses challenges for the accurate prediction of microbial protein functions [11]. Second, classical AI methods often perform well on specific datasets but exhibit weak generalization ability when faced with unseen data or slightly different data distributions [12, 13, 14], mainly limited by data heterogeneity, noise, and data distribution sensitivity [15]. Finally, classical AI methods also exhibit limitations when analyzing large‐scale datasets [16, 17].

Recently, large‐scale AI models [18] have been increasingly applied in various fields. For instance, AlphaFold2 [19] revolutionized protein structure prediction (the Nobel Prize in Chemistry 2022), whereas its successor, AlphaFold3 [20], extended predictions to protein complexes, nucleic acids, and ligands. Concurrently, Evo [21] enables multi‐omics sequence analysis/design, and BioBERT [22] and other pretrained language models [23, 24] significantly enhance biomedical text mining through domain‐specific pretraining. These models demonstrate strong potential for addressing the three core challenges outlined earlier by mining patterns from massive datasets to deliver precise analyses.

Several reviews have discussed the applications of large‐scale AI models in bioinformatics. For instance, Wang et al. [25] summarized the progress of ChatGPT in bioinformatics and explored its potential and challenges in areas such as omics and drug discovery. Zhang et al. and Chen et al. [26, 27] have examined the applications of transformer‐based language models in bioinformatics, and Shahid and Ruan et al. [28, 29] analyzed the challenges and limitations of fine‐tuning large language models (LLMs) in various bioinformatics domains. However, these reviews primarily focused on the applications of LLMs in bioinformatics and lacked a comprehensive summary of the applications of different types of large‐scale AI models across the key areas of bioinformatics.

**FIGURE 1:**
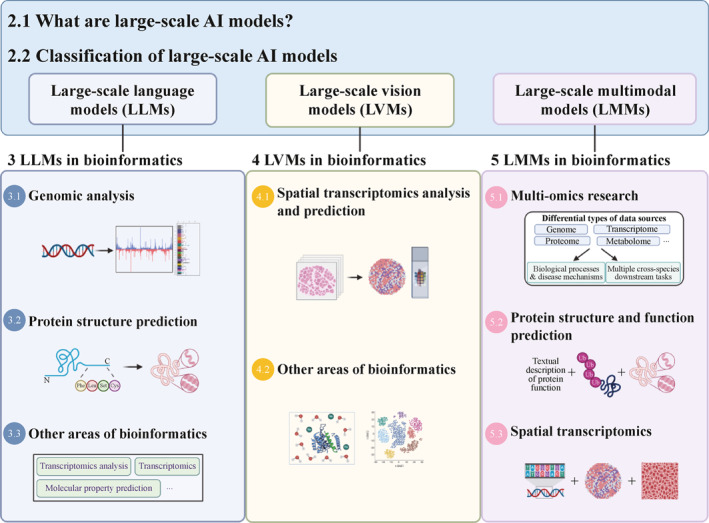
Main topics of the review.

Therefore, in this review, we first introduce the concepts and classifications of large‐scale AI models. Second, we summarize the practical applications of large‐scale AI models in bioinformatics (Figure 1). Third, future research directions and challenges are discussed. Overall, this review provides researchers with a comprehensive perspective to better understand the applications of large‐scale AI models in bioinformatics.

## 2 OVERVIEW OF LARGE‐SCALE AI MODELS

### 2.1 What are large‐scale AI models?

Large‐scale AI models refer to AI models with numerous parameters and complex architectures [18, 30, 31], which are typically trained on massive datasets and can handle complex tasks, such as natural language processing (NLP) and image recognition [32, 33, 34, 35]. Large‐scale AI models generally have characteristics such as numerous parameters, complex architectures, high computational resource demands, and strong adaptability to various tasks [36].

### 2.2 Classification of large‐scale AI models

Based on the pretraining data modalities [37] and task requirements [18], large‐scale AI models can be classified into three types: large‐scale language models (LLMs), large‐scale vision models (LVMs), and large‐scale multimodal models (LMMs) [37]. This classification is based on consensus in the field and the latest trends. Furthermore, the reasons for our choice of a modality‐based framework over other potential structures, such as organizing by biological problems or by model architecture, are as follows. On the one hand, a problem‐based structure would lead to significant redundancy, as a single model type such as LLMs finds applications across numerous biological domains. On the other hand, although an architecture‐based approach is technically precise, it is less intuitive for an interdisciplinary bioinformatics audience. Classification based on pretraining data modalities and task requirements not only provides a clear narrative, but also serves as a practical guide for researchers seeking to identify the most appropriate AI tools for their specific data types.

LLMs [38] are mainly used for processing and generating linguistic data and have shown strong performance and broad application potential across various NLP tasks. Recently, the rapid development of LLMs has primarily relied on the widespread application of the Transformer [39]. The Transformer comprises an encoder and decoder, both of which are composed of multiple stacked identical layers. Each layer contains two main components: self‐attention [39] and a feedforward neural network (FNN) [40]. Self‐attention computes the association weights between any position in a sequence, thereby overcoming the limitations of traditional neural networks in capturing long‐range dependencies. The FNN introduces nonlinear transformations, further enhancing the capacity of the model to express complex patterns. Through stacking, these components form a multilayered structure, enabling the Transformer to progressively construct and refine representations of the input sequences, thereby effectively processing various sequential tasks.

Building on this foundation, BERT [41] and GPT [42], two typical applications of the Transformer architecture, exhibit different design characteristics and applicable scenarios. BERT employs a bidirectional encoder structure, making it suitable for tasks that require a deep understanding of contextual information [43]. In contrast, GPT focuses on an autoregressive decoder structure that performs better in tasks that require generating coherent text [44].

LVMs [45] are primarily used to process image data, such as in image recognition and classification. LVMs combine the advantages of convolutional neural networks [46] and Transformers [47, 48, 49]. CNNs compute dot products in local regions using small‐sized convolutional kernels, focusing on extracting local features from images; however, they have limitations when processing long‐distance dependencies. As a complement, the self‐attention mechanism in Transformers dynamically computes correlations between different image segments, thereby capturing long‐range dependencies in images to understand the global connections between different regions. For instance, vision Transformers (ViTs) [50] convert various visual data into “visual sentences” [51] and perform next token prediction to achieve automated image understanding. After we contrast LVMs with LLMs, we found that LLMs typically rely solely on Transformer‐based architectures to process sequential data, whereas LVMs integrate CNNs’ local feature extraction with Transformers’ ability to capture long‐range dependencies to achieve comprehensive image understanding. This fundamental difference explains why LVMs are uniquely suited to tasks involving biological imaging, such as spatial transcriptomics (ST).

LMMs [52] enable unified understanding and generation of data by integrating information from different modalities. This process typically involves key components such as the feature extractor, fusion layer, and generator. First, the feature extractor extracts the corresponding feature representations from the input data of each modality. Next, the fusion layer performs cross‐modal interaction and integration of the extracted features. Finally, the generator generates the required output for the task based on the fused multimodal representations. For instance, the modality module in Macaw‐LLM [53] can simultaneously handle data from multiple modalities, such as text, images, and audio, thereby supporting multimodal understanding and interaction tasks.

## 3 LLMs IN BIOINFORMATICS

It is one of the basic studies in bioinformatics to employ various sequencing data (such as DNA, RNA, or protein sequences) to predict biomolecular structures and functions [54, 55, 56, 57, 58, 59, 60]. LLMs, particularly those based on the Transformer architecture, were originally designed to capture complex, non‐local contextual dependencies in natural language. Given the analogy between sequencing data and linguistic word sequences [61], LLMs can learn the underlying grammatical and semantic rules of biological sequences. Consequently, they have become increasingly prevalent in bioinformatics applications.

By leveraging architectures, such as Transformer to process large‐scale biological data, these LLM models play a pivotal role in critical bioinformatics tasks, including protein structure prediction and genome analysis. Because amino acid or nucleotide sequences are typically represented by single letters, LLMs cannot interpret these letters directly. Therefore, one of the key steps in applying protein sequences to LLMs for structural prediction is the transformation of sequencing sequences into numerical vectors.

**FIGURE 2:**
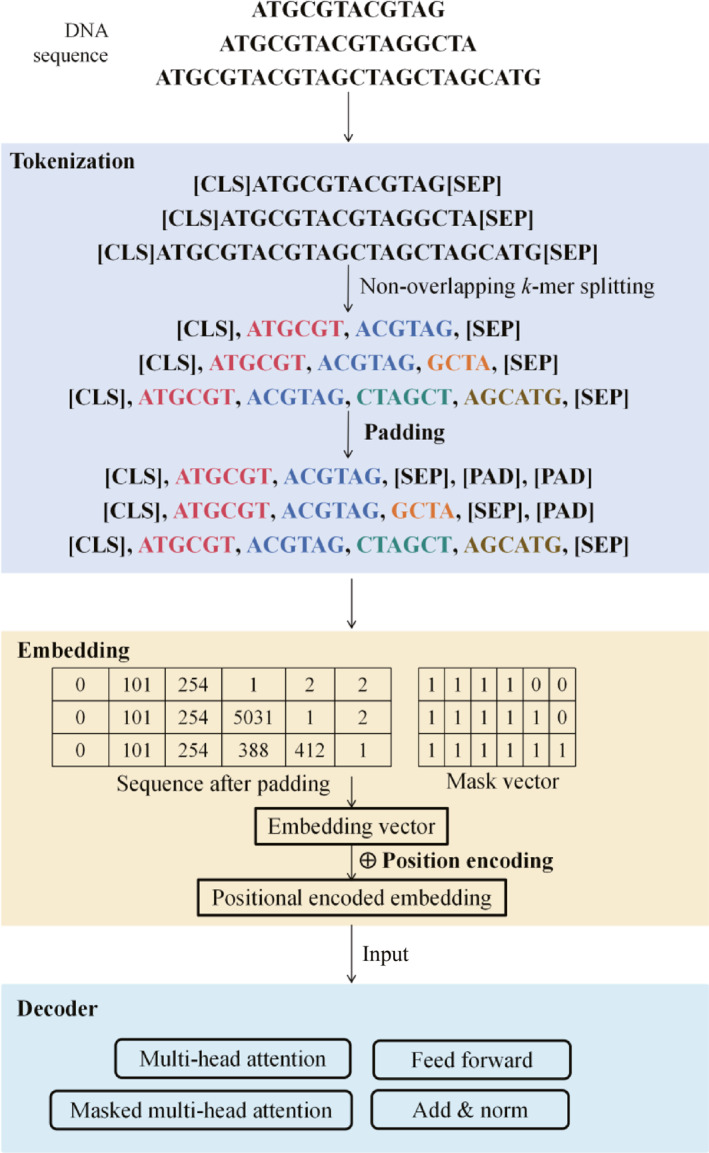
Main process of converting sequences into numerical vectors for analysis and prediction in LLMs (using DNA gene sequences as an example). Through tokenization, padding, and masking, the data undergo preprocessing while ensuring the model focuses exclusively on valid data. Subsequently, embedding operations map these sequences into vector spaces, combined with positional encoding to capture the relative positional information of nucleotides. The resulting numerical vectors are then input into the decoder for deep extraction of complex sequence features. DNA, deoxyribonucleic acid; LLMs, large language models.

Figure 2 uses DNA sequences as an example to illustrate the key steps in converting sequences into numerical vectors. First, tokenization is performed individually for each sequence, where the (CLS) and (SEP) tokens are added to mark the start and end of the sequence. Second, non‐overlapping *k*‐mer segmentation [62] (assuming *k* = 6) is applied to the sequence. For bases at the end of a sequence that form an incomplete *k*‐mer, the model typically treats it as a single token. These tokens are the basic units used by LLMs for understanding and prediction [63], and can be used to construct biologically meaningful vocabularies.

Next, embedding is used to map the padded and masked sequences into a vector space, similar to word embeddings [64] in NLP. To meet the requirements for uniform length when LLMs process sequences in batches, we not only extend shorter sequences to a uniform length by appending padding tokens (PAD), but also apply masking to distinguish between valid sequence elements and padding tokens, which can guarantee that the model focuses exclusively on valid data during prediction [65]. In addition, to capture the relative positions of nucleotides or amino acids within a sequence, positional encoding is typically added to the embedding vectors [66].

Finally, the numerical vectors are passed to the decoder for analysis and prediction. The FNN and attention mechanisms in the decoder are common to LLMs. However, each model selects more appropriate attention mechanisms based on practical applications, such as multi‐head attention mechanisms and multihead masked attention mechanisms. Among them, the multi‐head attention mechanism [67] divides input embeddings into multiple subspaces, with each self‐attention head independently focusing on different features. By computing multiple self‐attention subspaces in parallel, it captures various types of relationships. For instance, the decoder employs a multi‐head attention mechanism to capture diverse dependencies within the input data and employs a masking mechanism [68] to reduce the weights of future positions, ensuring that the model does not use future information during prediction. The synergistic interaction of each module ensures that the decoder generates accurate predictive results.

### 3.1 Genomic analysis

With the development of next‐generation sequencing technologies, the volume of genomic data has grown exponentially [69]. However, classical analytical methods exhibit an insufficient capability to extract features based on contextual information when dealing with complex genomic data [70, 71].

Consequently, LLMs, which leverage their deep understanding of complex sequences and automatic feature extraction capabilities [72], have been gradually applied to key tasks in genomics, such as gene expression and sequence function prediction [72]. Notably, genomes contain numerous non‐coding regions and repetitive sequences, and regulatory regions may be distant from the target genes. The diversity and noise in these data require stronger generalization capabilities, which increase the difficulty of sequence analysis [73].

Generally, the main steps for using LLMs in genomic analysis (Figure 2) are as follows. First, tokenization is performed individually on each sequence by adding tokens at the start and end positions and segmenting the sequence into multiple tokens to construct a vocabulary of gene sequences. The padded and masked sequences are then mapped to vector spaces through embedding. Subsequently, these numerical vectors are fed into the decoder, which employs self‐attention mechanisms to effectively capture contextual relationships between positions within the sequence. During the pretraining phase, LLMs typically adopt bidirectional context pretraining [41, 74], which considers the contextual information both preceding and following a given position in the sequence. This enables the model to identify and predict key regions within gene sequences, thereby enhancing the accuracy of subsequent specific tasks such as gene expression prediction and sequence function analysis.

Currently, bidirectionally context‐pretrained LLMs have been widely applied to gene expression and sequence function prediction [62, 75, 76, 77, 78, 79]. For instance, Enformer [75] employs an optimized self‐attention mechanism to integrate interaction information spanning up to 200,000 base pairs, thereby increasing the accuracy of gene expression prediction by 5% and 22.6% compared with the ML‐based ExPecto [80] in cross‐gene and cross‐tissue gene expression evaluations, respectively. DNABERT‐2 [62], by introducing byte pair encoding and linear bias attention (ALiBi), optimizes the handling of long sequences and outperforms HyenaDNA [77] across multiple tasks. The nucleotide Transformer (NT) [76] learns the context‐dependent features of nucleotides from unlabeled DNA sequences and applies them to various genomic prediction tasks through fine‐tuning. In promoter prediction tasks, NT achieves a correlation coefficient that is 10% higher than that of HyenaDNA, and in gene splicing prediction tasks, NT attained a correlation coefficient of 0.96, surpassing that of Enformer, which is 0.74.

### 3.2 Protein structure prediction

Protein structure prediction is one of the most important tasks in bioinformatics. Classical experimental techniques, such as X‐ray crystallography [81] and cryo‐electron microscopy [82], can provide highly precise structural information, but are usually costly and time‐consuming [83]. Recently, the application of large‐scale language models to protein structure prediction has attracted widespread attention. This section focuses on the application of sequence‐based LLMs in protein structure prediction, and Section 5.2 will discuss LMMs approaches that integrate multiple data sources.

LLM applications for protein structure prediction, such as for genomic sequences, are also based on sequence inputs. However, LLMs in the protein domains focus more on mapping sequences to structural spaces for protein structure prediction [84]; whereas in genomics, LLMs emphasize sequence pattern recognition and functional associations using their sequence modeling capabilities to process ultra‐long DNA sequences (Figure 2), as well as to capture and predict regulatory patterns [21].

The application of LLMs in protein structure prediction can be primarily categorized into multiple sequence alignment (MSA)‐based [85] and MSA‐free models [86], both of which are based on the Transformer architecture. All MSA‐based models require MSAs as input, whereas MSA‐free models typically operate on single sequences or their embeddings. Furthermore, MSA‐based models employ Transformer layers with row and column attention to extract co‐evolutionary signals from MSAs, enabling them to achieve high predictive accuracy for proteins with numerous homologs [85]. In contrast, MSA‐free models utilize multi‐head attention to generate contextual embeddings that capture structural and functional signals. However, the performance of MSA‐based models degrades significantly in the absence of sufficiently homologous sequences [19]. To date, MSA‐free models such as HelixFold‐Single [87] and ESM‐2 [88] have achieved competitive performance.

The main steps in using MSA‐free LLMs to predict protein structures include the transformation of protein amino acid sequences into numerical vectors through embedding. Subsequently, various computational models [89, 90, 91, 92, 93] facilitate feature fusion [94] by integrating embedding vectors with the physicochemical properties of amino acids (such as hydrophobicity and charge) to generate richer and more comprehensive feature representations. Following this integration, the decoder analyzes the integrated protein vector and predicts the protein structure. The predictive accuracy is ultimately enhanced through large‐scale pretraining [95] and fine‐tuning [96] methodologies applied to specific protein structure prediction tasks.

Currently, numerous LLMs have been successfully applied for protein structure prediction. For instance, ESM‐2 [88] focuses on sequence feature learning based on the aforementioned approach and is 60 times faster than AlphaFold in predicting 3D protein structures. xTrimoABFold [45] is specifically designed for predicting antibody structures and protein‐antibody interactions and outperforms AlphaFold2 [19] in both accuracy and computational efficiency for antibody structure prediction tasks.

### 3.3 Other areas of bioinformatics

In addition, LLMs have found applications in other important areas of bioinformatics, including transcriptomics [97], single‐cell sequencing analysis [98], and molecular property prediction. For instance, transcriptomics is used to study an organism’s transcriptome and the sum of all RNA transcripts. Transcriptomic analysis has enabled the study of changes in gene expression in different organisms and has been instrumental in understanding human disease [99, 100]. In transcriptomics, ERNIE‐RNA [101] introduces pairing constraints into the self‐attention mechanism, thereby enabling the capture of RNA structural features without MSA [102]. In single‐cell sequencing analysis, scFoundation [103] is pretrained on more than 50 million single‐cell transcriptomic datasets, making it widely applicable to tasks such as deep cell sequencing and cell drug response prediction. In molecular property prediction, SMILES‐BERT [104] learns deep representations of molecular structures through a masked SMILES recovery task, outperforming traditional methods based on CNNs (e.g., XGBoost [105]) and GCNs (such as HRGCN+ [106]) across multiple molecular property prediction tasks. MolecularGPT [107], based on LLaMA‐2 [108], is designed for few‐shot molecular property prediction through instruction fine‐tuning, and can handle more than 1000 tasks. In molecular zero‐shot learning, MolecularGPT improves accuracy by 15.7% compared with GIMLET [109] in classification tasks and reduces the root mean square error by 17.9% in regression tasks, demonstrating its strong generalization capabilities. In addition, in the field of scientific discovery, LLM4SD [110] can extract known information from scientific literature and uncover patterns from molecular data to predict molecular properties.

## 4 LVMs IN BIOINFORMATICS

With the development of bioinformatics, various experimental biological imaging datasets have been widely used. These images contain crucial biological information, such as cell morphology, tissue structure, protein localization, and molecular interactions, which are characterized by high dimensionality and complex correlations [111]. Local features and global patterns in biological images jointly determine biological significance, increasing the complexity of the analysis. Owing to their feature representation capabilities and ability to capture multi‐scale dependencies, LVMs can simultaneously model details at both the global tissue and local cellular levels. LVMs are increasingly applied in biological image analysis to improve the accuracy of prediction and classification in this field.

LVMs encompass several critical phases. Because the image data in bioinformatics typically have different resolutions and qualities, it is necessary to standardize the image size by resizing and normalizing the pixel values. The standardized images are segmented into fixed‐size patches, followed by dimensional transformation through embedding mechanisms, which collectively constitute the patch‐embedding process [109]. Positional encoding is subsequently incorporated into the embedding sequence before transmission to the decoder component. Within the decoder architecture, self‐attention mechanisms facilitate the computation of inter‐patch similarities, thereby capturing spatial relationships across distinct image regions. The implementation of multiple cascading encoder layers enables progressive feature extraction and representation optimization. The resultant feature representations extracted through LVMs after pretraining and fine‐tuning protocols can be applied across various downstream analytical tasks.

### 4.1 ST analysis and prediction

In ST experiments, RNA sequencing combined with microscopy or RNA imaging techniques [112] generates high‐resolution images. Classical ML methods [113, 114, 115, 116] require dimensionality reduction or feature selection when processing high‐dimensional image data, which is time‐consuming and prone to information loss [117]. LVMs can efficiently and accurately process high‐dimensional data using patching and self‐attention mechanisms. This model can generate global representations of cell populations or tissues with multiple layers of self‐attention mechanisms, thereby providing critical contextual information for the inference of gene expression levels and downstream tasks, such as transcriptomic data prediction.

For instance, Hist2ST [118] combines Transformer and graph neural network to analyze spatial dependencies and image features in histological images, achieving RNA ST data prediction from histological images. Its computational efficiency significantly exceeds that of the ML‐based ST‐Net [119] on the breast cancer dataset. THItoGene [120] combines ViT [50] with the Efficient‐CapsNet [121] neural network to capture long‐range dependencies and spatial hierarchies in images, which can predict gene expression data from histological images, and has achieved a performance score of 0.327 on the HER2+ dataset, surpassing that of Hist2ST, which is 0.250.

### 4.2 Other areas of bioinformatics

In addition, LVMs are gradually being applied in fields, such as protein function prediction and genomics. For instance, in protein function prediction, ViTScore [122] voxelizes the protein‐ligand interaction pocket into a 3D grid, where each voxel represents a spatial region of the protein structure and labels different types of atoms or amino acids. This not only preserves complete spatial information but also highlights the details of protein‐ligand interactions, which helps us accurately predict protein‐ligand docking poses. In genomics, GeneViT [123] converts gene sequences into numerical vectors and then maps these vectors into a two‐dimensional space using *t*‐SNE [124] method to generate images. ViT [50] is subsequently applied to extract image features for the classification of gene expression data. Its accuracy on benchmark datasets is 98.5%, surpassing the 96% accuracy of the ML algorithm MFOA [125].

## 5 LMMs IN BIOINFORMATICS

**FIGURE 3:**
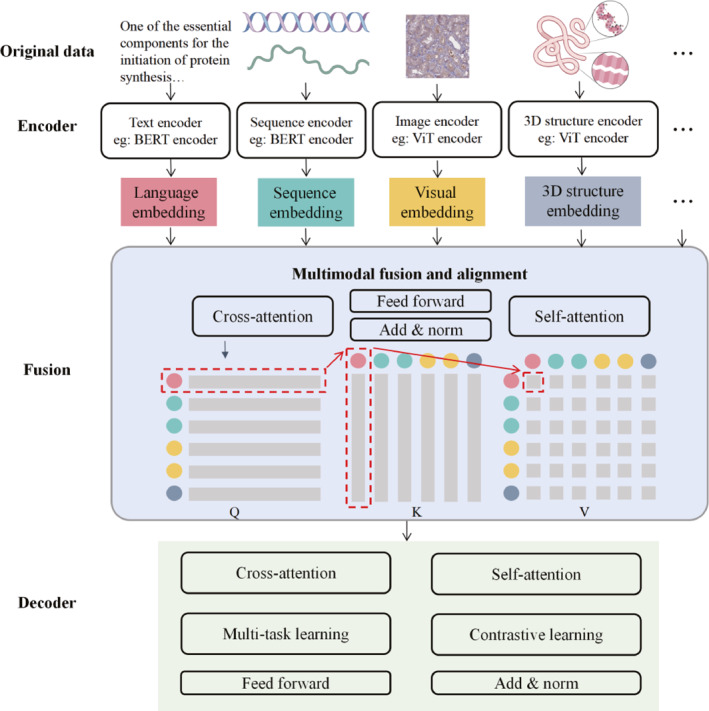
Main steps of multimodal data fusion for large‐scale multimodal models. Different modal data are transformed into unified representations through preprocessing and feature extraction. Appropriate fusion strategies can be adopted to integrate data, and decoders can dynamically weight and aggregate features from different modalities to increase fusion effects.

The field of bioinformatics involves multiple modal data types, such as genomic sequence data in text modality, transcriptomic data represented in matrix modality, and biological experimental images belonging to image modality, which are usually of high dimensionality, heterogeneity, and complex internal correlations [126]. Classical single‐modal models face inefficiency and serious information loss when handling multimodal data [127]. Because of their powerful data fusion and pattern recognition capabilities, LMMs provide a new technological solution for bioinformatics. The core of LMMs lies in fusing complementary information from different data sources to enhance the predictive capability [128]. Figure 3 illustrates the main steps of LMMs for different modal datasets. First, through their respective modal encoders, the data from the different modalities are preprocessed, and then converted into a unified representation after feature extraction for subsequent fusion and analysis. Second, a suitable fusion strategy (early fusion, mid fusion, or late fusion) [129] is used to integrate the data representations of different modalities whereas cross attention [130, 131] maps the features of each modality into three structures: query, key, and value, and each attention head is responsible for computing its own attentional scores. These scores reflect the relationship between themselves and different modalities. By dynamically weighting and aggregating the features of different modalities, it dynamically focuses on important information between different modalities, further improving the fusion effect and flexibility. Finally, LMMs are generally pretrained on large‐scale and diverse datasets to learn generalized feature representations. Strategies such as multi‐task learning [132] and contrastive learning [133] are employed during the training process to further increase the performance and generalization ability of the model. Subsequently, it is adapted to specific bioinformatics tasks through transfer learning or fine‐tuning.

### 5.1 Multi‐omics research

Multi‐omics research involves data from different omics to comprehensively understand biological processes and disease mechanisms [47, 70, 113, 116, 134]. LMMs can be used to discover deeper biological phenomena by integrating the information from different omics data. For instance, DeepOmix [46] provides new perspectives for understanding complex biological processes by integrating protein, genomic, and transcriptomic data, which demonstrates significantly better survival prediction performance than DL‐based survival models such as DeepSur [135] and DeepHit [136]. Evo [21] is able to parse and design DNA, RNA, and protein sequences, which performs well in predicting the effects of mutations on bacterial proteins and RNAs, as well as in modeling gene regulatory mechanisms. In the protein expression prediction task, the correlation coefficient of Evo reaches 0.60, far exceeding the 0.11 of GenSLMs [137].

Meanwhile, LMMs perform well in multiple cross‐species downstream tasks, providing new perspectives for understanding the relationships between gene expression and diseases among different species. For instance, the GeneCompass [138] model integrates 126 million single‐cell transcriptome data points from humans and mice, which not only predicts changes in cellular states but also reveals gene expression and regulation. The SATURN [139] model realizes the integration of non‐homologous genes across species by integrating scRNA‐seq data, cellular annotations, and protein expression from multiple species.

### 5.2 Protein structure and function prediction

In addition to unimodal, sequence‐based LLMs discussed in Section 3.2, multimodal data‐driven LMMs can also be used for protein structure prediction research. In protein research, common data modalities include amino acid sequences, 3D structures, experimental data, and textual descriptions. By joint learning from different modality data, we can better capture the complex properties and interaction mechanisms of proteins, thereby improving the comprehensiveness and accuracy of predictions [140, 141].

For instance, ESM3 [142] integrates the sequences, structures, and functions of proteins into embedding vectors in a unified way, effectively capturing both local and global structural and functional information. HelixProtX [120] integrates data from textual descriptions, amino acid sequences, and 3D structures, enabling the generation of any modality from any other. It outperforms LLMs such as Chroma [143] and HelixFold‐Single [87] in structure prediction. Released in 2024, AlphaFold3 [20] significantly outperforms AlphaFold2 [19] in predicting protein structures and protein–protein interaction structures by reducing reliance on MSA and introducing technical improvements such as diffusion modules. Chai‐1 [144] can be used to predict the molecular structures of proteins, small molecules, DNA, and other molecules without MSA. In protein antibody prediction, the accuracy without using MSA is 0.479, which is comparable to that when using MSA, and surpasses the accuracy of AlphaFold 2.3, which is 0.380. However, for protein monomer predictions, the accuracy of Chai‐1 without MSA is 0.852, and this is lower than that of AlphaFold 2.3, which was 0.903. Furthermore, by processing protein sequences, ligand information, and experimental constraint data, Chai‐1 achieved a success rate of 77% in the PoseBusters benchmark test [145], which is comparable to the success rate of 76% for AlphaFold 3.

### 5.3 Spatial transcriptomics

ST data involve various modalities, such as spatial information, gene expression data, histomorphological images, and even epigenetics data. Gene expression data not only depend on gene expression levels, but also closely relate to their spatial locations within tissues and specific cell types [146]. LMMs can effectively integrate information from different data sources and predict transcriptomic data by learning the relationships among different data modalities.

For instance, mclSTExp [147] uses hematoxylin and eosin stained images and ST data with spatial locations for multimodal comparative learning. It exhibited excellent performance in predicting spatial gene expression by combining intrinsic features with spatial contexts using self‐attention mechanisms. On the HER2+ dataset, mclSTExp outperforms BLEEP [148] by at least 23.01%. GHTNet [149] integrates different types of biological information, such as chromatin features, epigenomics data, and protein‐DNA interaction data. This integration enables better prediction of the binding specificity between transcription factors and DNA. Table 1 summarizes the representative large‐scale AI models used in bioinformatics.

**TABLE 1:** Representative large‐scale AI models applied in bioinformatics.

Model name	Year	Type	Main tasks	References
ESM‐2	2022	LLM	Protein 3D structure and function prediction	[88]
xTrimoABFold	2022	LLM	Antibody structure prediction	[45]
Enformer	2021	LLM	Protein 3D structure prediction	[75]
DNABERT‐2	2023	LLM	Genomic sequence classification and prediction	[62]
Nucleotide transformer	2024	LLM	DNA molecular phenotype prediction	[76]
ERNIE‐RNA	2024	LLM	RNA structural and functional predictions	[101]
scFoundation	2024	LLM	Drug response prediction and single‐cell perturbation prediction	[103]
Hist2ST	2022	LVM	Spatial transcriptomics data prediction	[118]
THItoGene	2024	LVM	Spatial gene expression prediction	[120]
Cellvit	2024	LVM	Segmentation of nucleus	[150]
SCTS	2023	LVM	Segmentation of nucleus	[151]
CellFormer	2023	LVM	Identify nucleus	[152]
ViTScore	2023	LVM	Protein‐ligand docking prediction	[122]
GeneViT	2023	LVM	Classifying cancerous gene expression	[123]
Evo	2024	LMM	Predict gene essentiality	[21]
GeneCompass	2024	LMM	Cross‐species biological investigations	[138]
SATURN	2024	LMM	Encoding genes’ biological properties	[139]
ESM3	2024	LMM	Protein 3D structure prediction	[142]
AlphaFold3	2024	LMM	Predict the structure and interactions of proteins, DNA, RNA, etc.	[20]
HelixProtX	2024	LMM	Protein sequence, structure and function prediction of proteins	[120]
Chai‐1	2024	LMM	Molecular structure prediction	[144]
mclSTExp	2024	LMM	Spatial transcriptomics expression prediction	[147]
GHTNet	2022	LMM	Predicting TF‐DNA binding specificity	[149]

Abbreviations: AI, artificial intelligence; LLM, large language model; LMM, large‐scale multimodal model; LVM, large‐scale vision model; TF‐DNA, transcription factor‐DNA.

## 6 CHALLENGES AND FUTURE PERSPECTIVE

In general, although large‐scale AI models have been increasingly used in various fields of bioinformatics, there are still many shortcomings and challenges that we need to address.

In terms of LLMs, LLMs have achieved great success in bioinformatics such as protein structure prediction. However, current protein structure predictions are mostly static [19, 20], and fail to reflect the highly dynamic nature and multiple conformations of proteins in organisms. Several studies [19] have attempted to employ LLMs to analyze protein dynamics; however, they performed poorly in identifying conformational fuzziness owing to insufficient dynamic information in the training data.

Therefore, one of the most important future research directions is to increase the predictive accuracy of protein dynamic conformations by increasing the ability of the model to learn the dynamic changes of protein conformations across states. Furthermore, how to balance and improve the accuracy and speed of large‐scale AI models in tasks, such as protein structure prediction and genetic data analysis, becomes a multi‐dimensional challenge. Relevant studies [153, 154, 155] have addressed this issue.

For example, parameter‐efficient fine‐tuning (PEFT, such as adapter and LoRA) can significantly reduce computational requirements during the fine‐tuning process. In protein function prediction tasks, PEFT can reduce the training time while maintaining prediction accuracy [153]. In genomic data analysis, adopting dynamic batching strategies can adaptively adjust batch sizes based on sample complexity, fully utilizing computational resources and improving analysis speed without sacrificing accuracy [154]. Additionally, specific optimizations for different hardware platforms (such as CPU, GPU, and TPU [155]) can further enhance the performance.

LVMs have been gradually applied in ST, particularly in the extraction and analysis of spatial structural information. However, annotation of ST data is often difficult and costly [156]. Therefore, one of the future research challenges is how to train efficient models using limited annotated data. Additionally, LVMs can be applied to analyze image data in genomics, such as chromosomal banding patterns or fluorescence images of chromosomes, to study chromosomal features or abnormal information [157], which may contribute to research on major diseases such as cancer.

In terms of LMMs, due to their ability in cross‐modal learning and feature fusion, LMMs are an important technological direction for parsing the interrelationships of multi‐omics data and thus elucidating the mechanisms of complex biological processes, owing to their ability in cross‐modal learning and feature fusion. However, the integration of multimodal information requires appropriate fusion techniques and timing.

In practical bioinformatics applications, achieving perfect matching of cross‐modal data is often challenging. How to process missing information, complete incomplete data, and resolve conflicting signals between modalities remain critical issues requiring urgent resolution. Recent studies have developed various strategies to address these challenges. For instance, feature fusion techniques (such as the multimodal feature fusion module [158]) can integrate complementary information between modalities to improve data completeness. For cross‐modal conflict issues, researchers have proposed commonality‐ and discrepancy‐sensitive encoders (CDS‐Encoder) and dynamic feature unification modules, which mitigate conflicts by capturing modal invariance and specificity, and dynamically integrating multimodal features, respectively [159, 160]. Furthermore, latent feature learning methods align the consistency and specificity between modalities through contrastive learning, effectively completing missing data and ensuring the spatial consistency of the cross‐modal data [161, 162].

Although these methods have demonstrated advantages in their respective fields, cross‐modal bioinformatics integration still lacks a systematic framework that balances modal generalization and specificity while improving the computational efficiency in practical applications.

It should be noted that while large‐scale AI models have provided powerful analytical and predictive capabilities for bioinformatics, challenges regarding model accessibility and reproducibility remain unresolved. These models typically demand substantial computational resources (GPU memory and training time), complex environmental configurations, and reliance on massive pretraining datasets. Concurrently, the increase in model parameters has led to an exponential increase in computational costs and energy consumption [163]. Addressing these issues requires multi‐dimensional coordination for model optimization. For example, the computational load can be reduced by optimizing the model architecture (e.g., model distillation [159] and compression [164]). Distributed computing (e.g., model slicing [165] and joint learning [166]) can be used to increase the throughput. With the maturation of model optimization technologies and new computing architectures [167], future research should focus on developing low‐resource, high‐performance bioinformatics models.

## 7 CONCLUSION

In summary, large‐scale AI models have exhibited great potential in bioinformatics. We systematically explored the burgeoning applications and transformative potential of LLMs, LVMs, and LMMs within a diverse bioinformatic landscape. However, challenges such as high computational resource demands, low generalization and interpretability, difficulties in cross‐modal data integration, and a lack of corresponding web services still remain.

Therefore, addressing how to improve the interpretability and generalization ability of large‐scale AI models through model optimization, develop corresponding algorithmic web services in combination with high‐performance computing technologies, or even provide low‐cost solutions, will be the main direction for future research on large‐scale AI models in bioinformatics.

## AUTHOR CONTRIBUTIONS

**Mingjing Li**: Conceptualization; investigation; visualization; writing—original draft. **Qichen Shang**: Investigation; validation; visualization. **Ziyang Dong**: Investigation; validation. **Zhixuan You**: Investigation; validation. **Le Zhang**: Funding acquisition; resources; writing—review and editing. **Ming Xiao**: Methodology; writing—original draft; writing—review and editing.

## CONFLICT OF INTEREST STATEMENT

The authors declare no conflicts of interest.

## ETHICS STATEMENT

This review article is based on a comprehensive analysis of existing literature and does not involve human or animal subjects, experimental research, or personal data collection.

## Data Availability

This review article does not generate any new data.
